# Survival in severe alpha-1-antitrypsin deficiency (PiZZ)

**DOI:** 10.1186/1465-9921-11-44

**Published:** 2010-04-26

**Authors:** Hanan A Tanash, Peter M Nilsson, Jan-Åke Nilsson, Eeva Piitulainen

**Affiliations:** 1 Department of Respiratory Medicine, Malmö University Hospital, Lund University, Malmö, 205 02, Sweden; 2 Department of Clinical Sciences, Malmö University Hospital, Lund University, Malmö, 205 02, Sweden

## Abstract

**Background:**

Previous studies of the natural history of alpha-1-antitrypsin (AAT) deficiency are mostly based on highly selected patients. The aim of this study was to analyse the mortality of PiZZ individuals.

**Methods:**

Data from 1339 adult PiZZ individuals from the Swedish National AAT Deficiency Registry, followed from 1991 to 2008, were analysed. Forty-three percent of these individuals were identified by respiratory symptoms (respiratory cases), 32% by liver diseases and other diseases (non-respiratory cases) and 25% by screening (screened cases). Smoking status was divided into two groups: smokers 737 (55%) and 602 (45%) never-smokers.

**Results:**

During the follow-up 315 individuals (24%) died. The standardised mortality rate (SMR) for respiratory cases was 4.70 (95% Confidence Interval (CI) 4.10-5.40), 3.0 (95%CI 2.35-3.70) for the non-respiratory cases and 2.30 (95% CI 1.46-3.46) for the screened cases. The smokers had a higher mortality risk than never-smokers, with a SMR of 4.80 (95%CI 4.20-5.50) for the smokers and 2.80(95%CI 2.30-3.40) for the never-smokers. The Rate Ratio (RR) was 1.70 (95% CI 1.35-2.20). Also among the screened cases, the mortality risk for the smokers was significantly higher than in the general Swedish population (SMR 3.40 (95% CI 1.98-5.40).

**Conclusion:**

Smokers with severe AAT deficiency, irrespective of mode of identification, have a significantly higher mortality risk than the general Swedish population.

## Introduction

Severe alpha-1-antitrypsin deficiency (AATD) is a hereditary condition characterised by low levels of AAT in serum and the lungs, a high risk of developing panacinar emphysema, and an increased risk of liver disease, primarily in early childhood and late adulthood [1-6]. Cigarette smoking is strongly associated with early development of emphysema in PiZZ individuals [2].

Previous studies of the natural history of severe AATD have indicated that it is a severe disorder leading to a considerably reduced life expectancy, and that emphysema is the most common cause of death [2]. However, our recently published study showed that PiZZ individuals, who have never smoked and have been identified by screening, do not have an increased mortality risk compared with the general Swedish population [7]. We also found that emphysema and liver diseases were the most common causes of death. Smokers were not included in the analysis.

The aims of this study were to analyse the mortality of PiZZ individuals, with respect to smoking habits and the mode of identification, to analyse the predictors of mortality, and further, to analyse the most common causes of death.

## Subjects and Methods

### Study population and data collection

The patients were selected from the Swedish National AATD Registry, which was started in 1991 [8]. Eligibility criteria for inclusion in the registry have been described previously [7]. Briefly, all PiZZ individuals in Sweden aged 18 years and over, are invited to participate in the registry. After inclusion, the patients are followed-up every 2 years by their attending physician. Smoking habits, symptoms, diagnoses, results of lung function and liver function tests are reported to the registry via a questionnaire.

The initial reasons for AAT analysis leading to the diagnosis of severe AATD were obtained from the questionnaire. The patients identified by the presence of respiratory diseases or symptoms, including repeated respiratory tract infections, are referred to as "respiratory cases" (R cases). The patients identified because of other diseases or symptoms are referred to as "non-respiratory cases" (NR cases). The NR cases include liver diseases, renal diseases, joint symptoms, repeated infections other than respiratory tract infection, high sedimentation rate, or other signs and symptoms for which plasma protein analysis has been performed as part of the clinical investigation. The patients identified by family/population screening are referred to as "screened cases" (S cases).

Details of smoking habits were also obtained from the questionnaire. Smoking status was based on the subjects' self-reports and was divided into two groups: smokers and never-smokers. A smoker was defined as any subject who had smoked more than one cigarette per day for at least one year, or more than 20 packs of cigarettes during his/her life. Smoking history was available for all cases.

Lung function tests were performed at local hospitals and were requested to be performed in accordance with European recommendations [9]. Forced expiratory volume in one second (FEV_1_) and forced vital capacity (FVC) were measured. The results of the lung function tests are expressed as the percentage of predicted values according to European reference tables [10]. The FEV_1_/FVC ratios are expressed as percentages. The results of the first spirometry at inclusion in the registry were analysed. Only pre-bronchodilator values were analysed, because a reversibility test with a bronchodilator was not consistently performed.

The date of death was obtained from the Swedish Registry of Deaths. Vital status was known for all cases at the closing point of the study (October 1^st^, 2008). The dates of lung and liver transplantations were obtained from the questionnaire and medical records. For each death, the medical records from the hospital at which the death occurred, were reviewed. These records included medical charts from the terminal hospitalization, last outpatient's visits (if the patient died at home) and the death certificate. An autopsy protocol for patients who had undergone complete post-mortem examination was obtained from The Department of Pathology at which the autopsy was performed. All available records and autopsy protocols were analyzed to determine the main causes of death. Records and autopsy protocols were also analysed concerning the occurrence of liver disease.

### Statistical analysis

Cumulative survival probabilities were estimated using the Kaplan-Meier method, and differences in survival were calculated with the log rank test. The period of the follow-up for the survival calculation was taken from the date of inclusion in the registry to the date of death, date of lung/liver transplantation or up to October 1^st^, 2008 (closing date of the study). Analysis of variance (ANOVA) was used for group comparisons. The Tukey Honest Significant Difference (HSD) test was used for multiple comparisons [11]. To compare death rates in the PiZZ individuals with the general Swedish population, a standardised mortality ratio (SMR) was calculated as the ratio of observed to expected deaths. The expected numbers of deaths were obtained using age-, sex- and date-specific death rates published in Sweden annually (Statistics Sweden) [12]. Confidence intervals for the SMR were computed from the Poisson distribution. The rate ratios for mortality were calculated as the ratio of the observed number of deaths per person year of follow-up to the expected death rate. These rate ratios are referred to as RR. The Cox proportional hazards model was used to examine the effects of baseline predictors on survival. The baseline predictors were gender, age at inclusion (in two categories), pre-bronchodilator FEV_1 _% of predicted (divided into four categories according to the GOLD stages), the presence of respiratory symptoms at inclusion. A p-value of < 0.05 was considered significant.

## Results

### Study population and lung function

Up to October 2008, a total of 1349 PiZZ individuals were included in the registry. Ten of them were excluded because of lung transplantation before being entered in the registry. The remaining 1339 individuals were included in the statistical analysis. The demographic baseline data and the results of lung function tests are shown in Table 1. The initial reasons for AAT analysis were: lung disease in 43% (R cases), liver disease and other diseases or symptoms in 32% (NR cases), and family/population screening in 25% of cases (S cases). Among the NR cases 20% were identified because of liver disease and 80% because of other diseases or symptoms. The number of years between the diagnosis and inclusion in the register ranged from 0.1 to 35 years. At the time of inclusion, respiratory disease or respiratory symptoms were reported in 38% of the NR cases and in 30% of the S cases. The S cases were younger than the R and NR cases at the time of diagnosis and inclusion. Lung function at inclusion was significantly lower in the R cases than in the NR and S cases (p < 0.001), while the difference between the NR and S cases was not significant. The mean follow-up time ranged from 0.1 to 17 years with no significant differences among the three groups.

**Table 1:** Demographic data of the study population and the results of lung function tests.

	All n (%)	R cases (n = 574)	NR cases (n = 431)	S cases (n = 334)
Men (%)	48	52	44	49
Smoking status				
Smokers, no. (%)	737 (55)	423 (74)	177 (41)	137 (41)
Never-smokers no. (%)	602 (45)	151 (26)	254 (59)	197 (59)
Respiratory symptoms at inclusion, no. (%)	839 (63)	574	166 (38)	99 (30)
Deaths, no. (%)	315 (24)	212 (37)	79 (18)	24 (7)
Lung transplant (no.)	56	51	3	2
Liver transplant (no.)	2	-	2	-
Mean (range) age at diagnosis (years)	41 (0-88)	51 (0.1-88)	40 (0.01-84)	24 (0-77)
Mean (range) age at inclusion (years)	47 (18-89)	54 (18-89)	45 (18-87)	35 (18-78)
Mean (range) follow-up time (years)	9 (0.02-17)	8 (0.02-17)	10 (0.02-17)	11 (0.07-17)
Mean age (range) at death	67 (20-95)	67 (30-95)	69 (20-90)	61 (37-85)
Mean (SD) FEV_1 _(% predicted)	72 (33)	50 (29)*	88 (25)	89 (23)
Mean (SD) FVC (% predicted)	84 (23)	75 (25)*	89 (18)	93 (16)
Mean (SD) FEV_1_/FVC ratio (%)	63 (23)	48 (22)*	75 (17)	76 (15)

R: respiratory. NR: non-respiratory. S: screened.

*p < 0.001 *vs. *NR cases and S cases.

Fifty five percent of the study population were smokers and most of them were identified by respiratory symptoms (57%). The mean (SD) number of pack years was 15 (13). The smokers were significantly older than never-smokers (p < 0.001). The never-smokers had significantly higher FEV_1 _than the smokers (p < 0.001), Table 2.

**Table 2:** Age and results of lung function tests in smokers and never-smokers.

	Smokers	Never-smokers
Men (%)	50	47
Mean (range) age at inclusion (years)	49 (18-84)*	44 (18-89)
Deaths, no. (%)	208 (28)	107 (19)
Mean (SD) FEV_1 _(% predicted)	59 (32)*	88 (25)
Mean (SD) FVC (% predicted)	80 (25)*	89 (18)
Mean (SD) FEV_1_/FVC ratio (%)	54 (23)*	75 (17)

*p < 0.001 *vs. *smokers and never-smokers.

Of 423 smokers in the R cases, 328 (78%) had emphysema, 61 (14%) chronic bronchitis, 11 (3%) asthma, 5 (1%) pulmonary fibrosis, 5 (1%) bronchiectasis and 13 (3%) had repeated respiratory tract infections. Of 151 never-smokers in the R cases, 71 (47%) had emphysema, 32 (21%) chronic bronchitis, 26 (17%) asthma, 4 (3) pulmonary fibrosis, 6 (4%) bronchiectasis and 12 (8%) repeated respiratory tract infections.

## Survival

**Figure 1:**
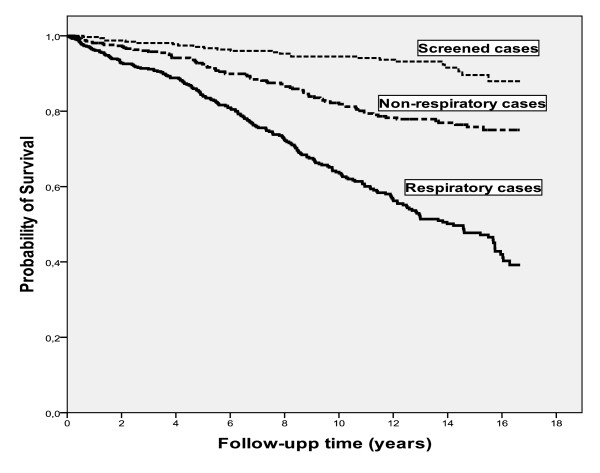
**Kaplan-Meier analysis of mortality stratified by mode of identifiaction in 574 Respiratory cases, 431 Non-respiratory cases, and 334 Screened cases**. The analysis showed a significant difference in survival time between R cases, NR cases and S cases, with estimated median survival times of 12 years, 14 years and 16 years, respectively (p < 0.001).

**Figure 2:**
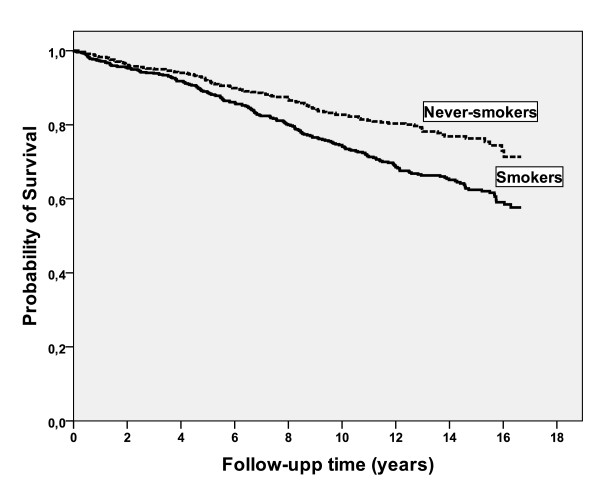
**Kaplan-Meier analysis of mortality stratified by smoking habits in 737 smokers and 602 never-smokers**. The analysis showed a significant difference in survival time between the smokers and the never-smokers, with estimated median survival times of 13 years and 14 years, respectively, (p < 0.01).

Fifty six patients received a lung transplant and two patients received a liver transplant; their mortality data are censored at the date of transplant. During the follow-up time 315 (24%) patients died. The median survival time for the whole study population since inclusion was 14 years, which was estimated by the Kaplan-Meier method. When the Kaplan-Meier analysis was stratified by mode of identification, there was a significant difference in survival time between R cases, NR cases and S cases, with estimated median survival times of 12 years, 14 years and 16 years, respectively, (p < 0.001), Figure 1. The Kaplan-Meier analysis was also stratified by smoking habits, Figure 2. The estimated median survival time for the smokers was significantly lower than the never-smokers, 13 and 14 years, respectively, (p < 0.01).

The SMR for the whole study population was 3.80 (95% CI 1.80-2.30). Gender-specific SMRs were 3.80 (95%CI 3.20-4.40) for men and 3.90 (95% CI 3.30-4.60) for women.

The smokers had a higher mortality risk than never-smokers, with a SMR of 4.80 (95%CI 4.20-5.50) for the smokers and 2.80 (95%CI 2.30-3.40) for the never-smokers. The Rate Ratio (RR) was 1.70 (95% CI 1.35-2.20). Stratifying the SMR by the mode of identification, showed that the SMR was 4.70 (95% CI 4.10-5.40) for the R cases and 3.0 (95%CI 2.35-3.70) for the NR cases. It should be noted that the SMR for the S cases was also significantly over 1, with 23 deaths and 9.9 expected (SMR 2.30 (95% CI 1.46-3.46)).

To assess the impact of bias by an interaction between the smoking habits and mode of identification, the SMR was calculated for the smokers and never-smokers in the R, NR and S cases, Table 3. Among the R cases, the smokers had a significantly higher mortality risk than the never-smokers. The RR was 1.80 (95% CI 1.30-2.50). Also for the S cases, the mortality risk of smokers was significantly higher than the general Swedish population.

**Table 3:** Standardised mortality ratios of smokers and never-smokers stratified by mode of identification.

	Smokers		Never-smokers		
	**O/E**	**SMR (95% CI)**	**O/E**	**SMR (95% CI)**	**RR (95% CI)**

Respiratory cases	156/27.20	5.70 (4.90-6.70)	57/18.20	3.10 (2.37-4.06)	1.80(1.30-2.50)

Non-respiratory cases	35/11.20	3.10 (2.20-4.34)	44/15.40	2.85 (2.07-3.83)	1.10(0.70-1.70)

Screened cases	17/5	3.40 (1.98-5.43)	6/5	1.20 (0.44-2.63)	2.80(1.10-10.40)

O: Observed number of deaths.

E: Expected number of deaths from the general population.

CI: Confidence interval.

RR: Rate ratio.

In the multivariate Cox model of mortality, including 1309 individuals with complete data on all predictors, i.e. age over 50 years, FEV_1_% of predicted below 80% and the presence of respiratory symptoms at inclusion, were significantly associated with shorter survival time, Table 4.

**Table 4:** Multivariable Cox Regression Models for Survival in PiZZ Individuals*

	No.	Risk Ratio	95%CI	P value
Gender				0.90
Male	635	1.00	0.80-1.30	0.90
Female	674	1.0		
Age at inclusion, years				<0.001
>50	580	5.50	4.0-7.50	<0.001
≤50	729	1.0		
Pre-bronchodilator FEV_1 _% of predicted				<0.001
	183	4.30	2.90-6.40	<0.001
< 30	230	2.00		<0.001
30-49	263	1.60	1.40-3.00	0.020
50-79	633	1.0		
≥ 80			1.10-2.40	
Respiratory symptoms at inclusion^&^				0.001
Yes	820	2.20	1.40-3.50	0.001
No	489	1.0		

** 1309 individuals with complete data on included predictors.

^&^irrespective of mode of identification.

## Causes of death

Medical records were available for 302 (96%) decedents. Autopsies were performed in 55 cases (18%). Autopsy protocols were available for all of these cases. The main causes of death were respiratory diseases in 175 (58%), liver diseases in 37 (12%) and other diseases in 90 (30%), Table 5. The 29 malignancy complications included 6 pulmonary carcinoma, 8 urinary-genital carcinoma, 6 gastrointestinal malignancy, 3 melanoma, 2 lymphoma, 2 pancreas carcinoma and 2 breast carcinoma.

**Table 5:** The main causes of death in 302 decedents.

	All N = 302 n (%)	Smoker N = 197 n (%)	Never-smokers N = 105 n (%)
Respiratory	175 (58)	134 (68)	41 (39)
Respiratory failure	117	92	25
Pneumonia	48	34	14
Pneumothorax	10	8	2
Hepatic	37 (12)	15 (8)	22 (21)
Liver failure	13	7	6
Primary liver carcinoma	18	7	11
Gastrointestinal bleeding	4	-	4
Intra-abdominal infection	2	1	1
Other	90 (30)	48 (24)	42 (40)
Malignancy complication	29	14	15
Cardiovascular accident	23	14	9
Cerebrovascular accident	14	6	8
Intra-abdominal infection*	8	6	2
Gastrointestinal bleeding*	2	1	1
Pulmonary embolism	6	4	2
Suicide/accident	5	3	2
Renal failure	3	-	3

* without known liver disease

## Discussion

In this study, the survival is presented for a large number of adult PiZZ individuals who have been followed up prospectively. The results confirm that PiZZ smokers, irrespective of mode of identification, have a significantly higher mortality risk than the general Swedish population. Our results show the importance of early detection of AAT deficiency. All individuals with severe AAT deficiency should be identified before they reach the age of their smoking debut.

When the individuals were stratified by mode of identification, and the survival of smokers and never-smokers was analysed, we found that even among the screened cases, smokers had a reduced survival time compared with never-smokers. However, also among the R cases smokers had a significantly higher mortality risk than never-smokers, with a SMR of 5.70 and 3.10, respectively. Our results suggest also that increased mortality was independently associated with higher age, lower FEV_1 _and the presence of respiratory symptoms at inclusion, Table 4.

The current study extends previous studies regarding mortality in PiZZ individuals by comparing the survival of a large number of PiZZ individuals [2,13-17]. The three groups R cases, NR cases and S cases differed with respect to smoking habits, age at inclusion and lung function (Table 1). The NR cases and the S cases seem to have normal lung function, but at the time of inclusion 38% of the NR cases and 30% of the S cases had respiratory symptoms, because in many cases the diagnosis was made a long time before their inclusion in the registry.

Previous studies on the natural history of AATD have been strongly influenced by ascertainment bias due to the fact that AAT analysis is most often performed in subjects with respiratory symptoms [13]. Therefore, many asymptomatic PiZZ individuals escape detection. The earliest study on the natural history of AATD, published by Larsson, indicated that it is a serious disorder associated with a poor prognosis [2]. He determined the cumulative probability of survival in 248 PiZZ subjects and found it to be significantly reduced compared with the normal Swedish population. In his study the median survival time was only about 40 years for smokers, and 65 years for never-smokers. However, the study population was highly selected. Ninety percent of the patients were identified because of symptoms and the majority were smokers. Another study of 124 AAT-deficient patients published by Brantly showed the cumulative probability of survival to age 50 of 52% and only a 16% chance of surviving to 60 years of age [14]. The majority of the individuals in this study were men, ex-smokers who had become dyspnoeic between 25 and 40 years of age. Because of the selection bias in both of these studies, the estimated life expectancy was probably too pessimistic. Recently, some studies have reported long-term survival and causes of death [15-17]. Seersholm analysed the life expectancy of 255 non-index patients who did not have pulmonary symptoms [15]. As in the current study, the survival of smokers in non-index cases was less than that of never-smokers and never-smokers did not have an excess mortality compared with the normal Danish population. However, this study did not show any difference in survival between smokers and never-smokers in index cases. The study included only a limited number of never-smokers and used a different approach for the survival calculation. In another study, Seersholm reported that FEV_1 _was a major risk factor for mortality in patients with severe AAT deficiency, stating that the median survival time for patients with FEV_1 _below 25% of predicted was only 6.3 years [16]. A mortality study recently published by Stoller *et al. *showed that severe airflow obstruction was a major determinant of mortality. Increasing age, lower FEV_1 _and lower education level were also associated with higher mortality [17]. The most common underlying causes of death were emphysema (72%) and liver cirrhosis (10%). This study compared the clinical features between decedents and survivors in the U.S. registry, but no comparisons between smokers and never-smokers were made. Neither respiratory nor other symptoms were reported, nor was the mode of identification. The SMR was stratified by quintiles of initial postbronchodilator FEV_1_, not by smoking habits or mode of identification, In contrast to our study, primary liver carcinoma was not found as a cause of death in the study by Stoller *et al.*

The natural history of AATD is incompletely known, although more than 40 years have elapsed since the deficiency was discovered. The selection in the Swedish national AAT registry is less skewed than in other registries in which most of the patients are identified because of respiratory symptoms. In the Swedish AAT registry, the majority of subjects is identified by reasons other than respiratory symptoms, and includes a large number of never-smokers. However, only 25% of the individuals were identified by family/population screening, and the remainder was identified by a disease or symptoms. Furthermore, although the detection rate of AATD is relatively high (20%) in Sweden, the majority of adult PiZZ individuals remains unidentified, and their health status is unknown, which is one limitation of our study. Another limitation is that we reported lung function as pre-bronchodilator FEV_1_, because a reversibility test was not performed in all cases. The third limitation is that we compared the SMR for the study population with the SMR for the general Swedish population, because no data are available on mortality in the subgroups based on smoking habits in the general Swedish population.

## Conclusion

We conclude that PiZZ smokers, irrespective of mode of identification, have a significantly higher mortality risk than the general Swedish population. Early detection of AAT deficiency is important in order to make it possible to implement protective measures such as inhibiting smoking.

## Competing interests

The authors declare that they have no competing interests.

## Authors' contributions

HT collected and analysed the data and developed the method to calculate the survival by SMR. She collected and reviewed the medical records and death certificates to determine the causes of death. She has written manuscript. EP, the main supervisor, is responsible for the Swedish AAT registry. She helped with advice throughout the study, and participated in the examination of the medical records to determine the cause of death.

JÅN, statistician, gave statistical advice, helped with the calculation of SMR, and in selecting the right statistical tests. PN is assistant supervisor. He contributed with medical and statistical advice. All authors read and approved the final manuscript.
